# Age as a moderator of the relationship between planning and temporal information processing

**DOI:** 10.1038/s41598-022-05316-6

**Published:** 2022-01-28

**Authors:** Katarzyna Jablonska, Magdalena Stanczyk, Magdalena Piotrowska, Aneta Szymaszek, Barbara Lukomska, Hanna Bednarek, Elzbieta Szelag

**Affiliations:** 1 Faculty of Psychology, SWPS University of Social Sciences and Humanities, Warsaw, Poland; 2 Laboratory of Neuropsychology, Nencki Institute of Experimental Biology of the Polish Academy of Sciences, Warsaw, Poland

**Keywords:** Cognitive ageing, Problem solving

## Abstract

Planning is a fundamental mental ability related to executive functions. It allows to select, order and execute subgoals to achieve a goal. Studies have indicated that these processes are characterised by a specific temporal dynamics reflected in temporal information processing (TIP) in some tens of millisecond domain. Both planning and TIP decline with age but the underlying mechanisms are unclear. The novel value of the present study was to examine these mechanisms in young (*n* = 110) and elderly (*n* = 91) participants in Tower of London task, considering two structural properties of problems: search depth related to static maintenance in working memory, and goal ambiguity reflecting dynamic cognitive flexibility. Results revealed that TIP predicted planning accuracy both directly and indirectly (via preplanning) but only in young participants in problems characterised by high goal ambiguity. Better planning is related to longer preplanning and more efficient TIP. This result demonstrates for the first time age-related differences in the contribution of TIP to planning. In young participants TIP contributed to dynamic cognitive flexibility, but not to static maintenance processes. In elderly such relation was not observed probably because the deficient planning might depend on working memory maintenance rather than on cognitive flexibility.

## Introduction

The ability to plan short-term activities, in addition to inhibition, initiation, shifting, monitoring, and flexibility, is one of the core components of executive functions, which provide higher order conscious control of human behaviour [e.g.,^1,2^]. Planning involves mental identification of specific goals and subgoals, including the simulation and anticipation thereof, in order to select the appropriate sequence of acts to achieve an ultimate goal^3–5^. These mental operations precede the efficient execution of goal-oriented actions. Neuropsychological and neuroimaging evidence has suggested that dorsolateral and orbital prefrontal regions play a crucial role in executive functions, including mental planning^6^.

Current data indicate that these functions undergo age-related deterioration, which is believed to be associated with structural and functional changes in the prefrontal cortex in advancing age^7–11^. However, many open questions remain about the specific mechanisms and processes underlying these age-related declines and the neural mechanisms underlying deficient planning in elderly are still poorly understood.

Many studies on planning have been based on various versions of the Tower of London task^12^, including the Freiburg version (TOL**–**F) used in the present study. This task consists of a set of coloured balls on three pegs that the participant is asked to move, one at a time and from peg to peg, from a start state to a goal state. Thus, it requires the mental planning of a sequence of moves, where participants are free to select their own trajectory of acts. Planning accuracy (PA) is measured by the number of problems solved in the minimum number of moves (i.e., the optimal solution). Performance of the task comprises two phases: the mental preparation phase (i.e., time prior to any movement execution, quantified as the initial thinking time; ITT) and the execution phase (i.e., after the first ball has been picked up). While the former phase reflects preplanning, the latter, in addition to the performance of moves, also relies on online planning. To find the optimal solution, one should construct a plan during the first phase and, then, perform the planned moves to achieve the goal during the second phase. Studies have shown that longer ITT is generally related to better PA^13^. Elderly participants often demonstrate shorter ITT (reflecting shorter preplanning) than young participants; moreover, they have greater problems with updating during execution and commit more mistakes in the mental preparation phase due to the reduced stimulus support and greater working memory load^14^.

Numerous reports indicate that the overall PA measure may be misleading because the structural properties of the task, in addition to the number of moves, critically affect performance [e.g.,^15^]. Therefore, in this study we aimed to explain age-related changes in planning, taking into account two structural properties of TOL**–**F—goal ambiguity (GA) and search depth (SD)—across problems with a minimum number of four to six moves^16^. According to Köstering et al.^11^, GA *“…refers to the ambiguity with which the sequence of final moves is derivable from the mere configuration of the goal state*”. It corresponds to the cognitive flexibility that is most engaged in solving problems with an ambiguous goal hierarchy (i.e., where the order of the subgoals cannot be straightforwardly inferred from the goal state). The solution of such problems requires a search across a wide problem space, considering several alternative sequences of moves, switching between them, and choosing the optimal one^11^ (for more explanations see also the Methods section below and Fig. 2). In contrast, SD *“…refers to the number of intermediate moves to be considered before execution of the first goal move”*^11^. Greater SD requires more working memory capacity, as one searches not across the breadth of a problem space, but rather explores a specific solution path in depth^11^ (see also below). Each TOL**–**F problem may be characterised by its levels of GA or SD being either high or low. These two properties allow TOL**–**F problems to be categorised in four ways: (1) high GA and high SD (GA_High_ SD_High_); (2) high GA and low SD (GA_High_ SD_Low_); (3) low GA and high SD (GA_Low_ SD_High_); and (4) low GA and low SD (GA_Low_ SD_Low_).

Each of these categories is characterised by a unique combination of cognitive resources and may involve working memory capacity and cognitive flexibility to various extents^5,17–19^. The influence of structural properties can help explain the mixed evidence for the engagement of various executive functions in TOL**–**F performance, such as inhibition, set-shifting, and working memory processes^13,20^.

It is commonly accepted that planning is not a single cognitive activity, but rather involves the interplay of multiple mental operations. The organisation of planned acts is characterised by the precise and flexible control of sequential movements over a short time period. Its dynamic temporal structure facilitates the ability to predict and anticipate a trajectory of mental operations in order to plan and organise sequences of actions which should be performed in order to achieve the intended goal.

The main objective of the present study was to understand individual differences and age-related differences in planning with reference to temporal information processing (TIP) using the aforementioned structural properties (GA and SD). The rationale for this approach comes from a number of literature studies—including studies conducted at our laboratory—that indicate that TIP in the millisecond range sets a frame for our mental activity and is linked to general principles of cognition^21–28^.

It has been long known that TIP is not a monolithic entity and several time ranges or processing windows may be distinguished. The present study is focused on millisecond level related to analytical sequential mental operations in which consecutive elements within incoming events are identified to be, next, processed successfully. For these effective operations temporal resolution is of crucial importance. It can be measured by a minimum time gap between stimuli presented in rapid sequences which is necessary for a subject to identify their order (i.e., relation before-after) correctly. Previous studies revealed that in young subjects such a gap has a duration of some tens of milliseconds with strong inter- and intra-individual variability^23,24^. Usually, shorter gap reflects better temporal resolution and corresponds to more efficient processing of incoming information. The efficiency of such temporal resolution may be measured with the temporal-order judgement task which was applied in the present study.

Previous studies have indicated that many mental functions (e.g., attention, working or short-term memory, language, or executive functions) can be characterised by a specific temporal dynamics, therefore, efficient TIP is crucial for human cognition and sets a frame for many mental functions^25–28^. This notion is supported by neuroimaging data which indicated that TIP involves the interaction of multiple brain areas, including both task-independent core timing structures and task-related areas activated in a context-dependent fashion [e.g.,^29–31^]. The core timing network is supposed to be an essential node of different networks involved in time processing and engages mainly supplementary motor area and the basal ganglia.

Another evidence for the link between TIP and cognitive functions comes from aging studies. It has been shown that deterioration in TIP, including deficient temporal resolution, accompanies age-related cognitive declines^25,28^. For example, in our previous report^26^, using the Tower of London—Drexel University version, we indicated that aging may be characterised by the overlapping of deteriorated temporal resolution and deteriorated executive functions reflected in the index of total move score. Furthermore, in the report by Jablonska et al.^32^, using both behavioural and neuroimaging data, we indicated that TIP supports the differentiation between processes engaged in working memory assessed with the n-back task. We found evidence for a strong contribution of TIP to manipulation, which requires the constant reorganizing and updating of processed material. This association was not observed for more static maintenance processes that rely to a greater extent on information storage.

On this basis, one may expect that, because of the temporal dynamics of mental operations during planning, they may be rooted in a defined millisecond template reflected by the temporal resolution that creates the neural frame for our mental activity, including executive functions. Hence, patterning in time might be the characteristic feature of efficient mental planning and may explain age-related deterioration in these processes. Considering these rationale, TIP on millisecond range may be considered as a logistic brain function that facilitates the ability to predict and anticipate events, as well as to organise and coordinate sequences of events.

**Figure 1:**
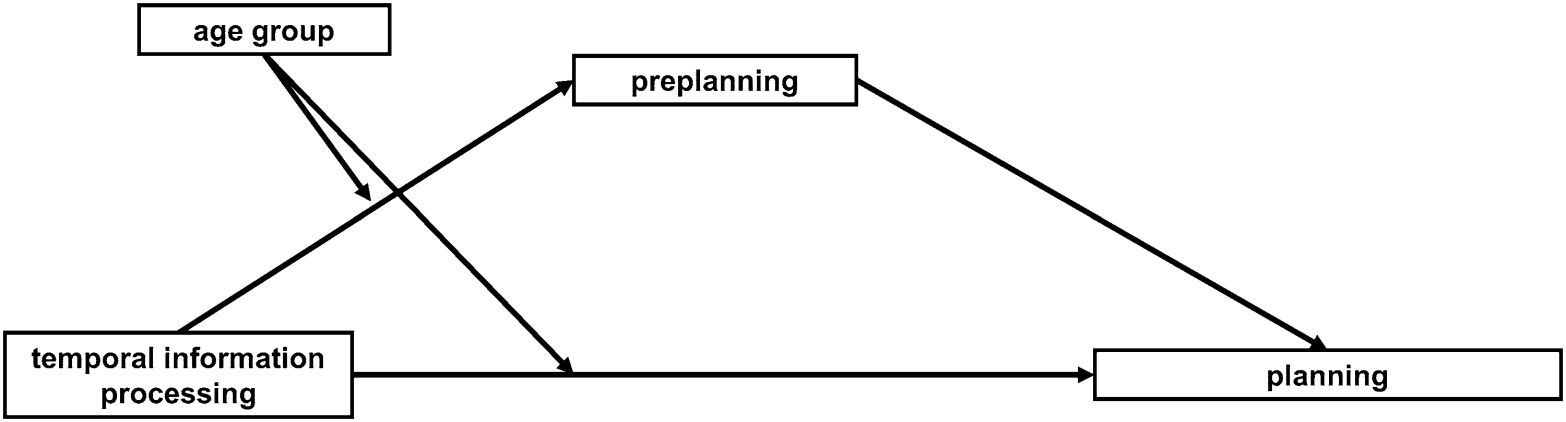
Conceptual model of the complex interrelations between millisecond temporal information processing (TIP) and planning, verified in particular categories of TOL**–**F problems.

In this study we tested the hypothesis that age moderates the relationship between planning and TIP using conditional process analysis^33^. Moreover, in the proposed model (Fig. 1), we tested the role of preplanning (measured with ITT) as a mediator of the relationship in each of the aforementioned four categories of TOL**–**F.

We assume that skilled TIP (reflected in higher temporal resolution) influences TOL**–**F performance in two ways. Firstly, it supports the effective use of the preplanning (reflected in longer ITT) by improving flexible switching between alternative subgoal hierarchies, allowing one to choose the optimal solution path during the preplanning phase. This may be more distinct in young adults, who rely more often on the more effective preplanning strategy. In elderly participants, this effect may be weaker.

Secondly, TIP directly facilitates better performance, as it enables flexible adjustments during the execution phase. Indeed, one may hypothesise that problems characterised by GA_High_ require more cognitive flexibility and dynamic manipulations in working memory than GA_Low_ problems. In such problems, therefore, skilled TIP might be expected to play a greater role. In contrast, problems characterised by SD_High_ require rather the cognitive stability involved in working memory maintenance, as opposed to cognitive flexibility and manipulation. Hence, TIP might be expected to play a lesser role in SD_High_ problems than in GA_High_ problems.

To summarise, the aim of the present study was to verify the association between planning and TIP in young and elderly adults, considering the categories of problems in TOL**–**F, in order to explain where and how age-related changes emerge.

## Methods

### Participants

The sample consisted of 201 participants classified into two age groups: a young group aged from 19 to 29 years (*n* = 110; *M* ± *SD* = 23.3 ± 2 years; male:female = 65:45) and an elderly one aged from 61 to 77 years (*n* = 91; *M* ± *SD* = 67.6 ± 3.8 years; male:female = 81:10). They were recruited via social media and adverts in local newspapers.

All participants were right-handed, had normal hearing verified by pure-tone screening audiometry (Audiometer MA33, MAICO), and had no systemic diseases or neurological or psychiatric disorders. Moreover, the elderly candidates were screened for cognitive impairment with the Mini-Mental State Examination^34^ (an inclusion criterion was scoring at least 27 points) as well as for depression with the Geriatric Depression Scale^35^ (an inclusion criterion was scoring between 0 and 5 points). All participants gave written informed consent prior to the study. In this study we present data obtained in three projects. These studies were in line with the Declaration of Helsinki and were approved by: (1) Bioethics Committee of the Nicolaus Copernicus University (permission no. KB 289/2019); (2) Senate Ethical Commission at the University of Social Sciences and Humanities (Permission No. 1/2017); (3) Research Ethics Committee at the Faculty of Psychology, University of Social Sciences and Humanities (permission no. 7/2019).

### Procedure

Each participant completed two experimental tasks during individual sessions: (1) the Tower of London—Freiburg version (TOL**–**F)^36^ to assess planning and (2) the Temporal-order judgement task to assess the efficiency of TIP^37^. Both these procedures were conducted in a sound-proof room at the Nencki Institute of Experimental Biology.

#### Tower of London task

To measure the effectiveness of planning, we used the TOL**–**F; it is a standardised computerised version of the TOL task implemented in the Vienna Test System (VTS). The task comprises 24 problems solved by each participant individually. Each problem consists of a start state presented in the lower part of the computer screen and a goal state presented in the upper part of the screen. Both the start and goal state consist of a picture of three balls (red, yellow and blue) placed on three rods of different heights that may hold either one, two, or three balls. The task is to transfer the balls from the start state to the pre-defined goal state in the minimum number of moves following three rules: only one ball can be moved at a time; balls cannot be placed outside the rods; and if more than one ball is stacked on a rod, only the topmost ball can be moved. There are 8 four-, five-, and six-move problems presented in increasing difficulty.

There was an instruction phase prior to the testing phase, in which the understanding of the task was verified by 2 two-move problems and a practice phase with an additional set of 4 three-move problems. During the testing phase, the participant had one minute to solve each problem. If this time limit was exceeded three times in a row, the test was automatically aborted. Participants were instructed to always plan ahead before starting to move the balls and to try to solve each problem as quickly and as accurately as possible in the minimum number of moves. The following two outcome measures were analysed:

- PA, defined as the number of problems solved in a minimum number of moves within the time limit; and
- ITT, defined as the mean time distance between the initial presentation of the problem and the first move.

**Figure 2:**
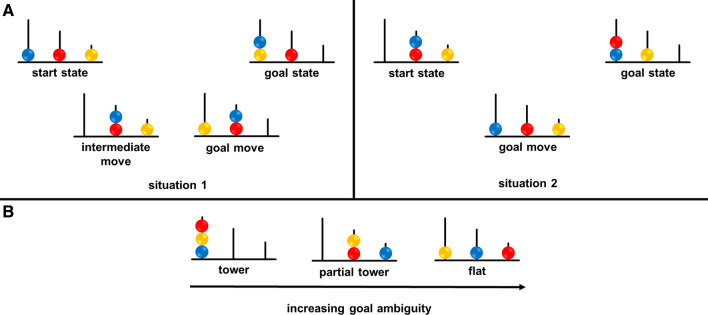
Schematic representation of two structural properties of TOL-F problems. (**A**) Search depth (SD) is reflected in the number of intermediate moves necessary to achieve the first goal move. In situation 1, the first goal move is placing the yellow ball on the tallest rod. To achieve this, the participant must first perform one intermediate move (place the blue ball on the middle rod), after which the goal move becomes possible. In situation 2, the first goal move is placing the blue ball on the tallest rod. No intermediate move needs to be performed to enable this move, therefore, the first goal move can be performed immediately. (**B**) Goal ambiguity (GA) is determined by the configuration of the goal state (indicated by an arrow). The tower goal state is characterised by the lowest ambiguity. One can straightforwardly derive the order of goal moves from the configuration itself—first, the blue ball has to be placed in its goal position, then the yellow ball, and finally the red ball. The flat configuration is characterised by the highest ambiguity. The configuration itself does not give any information about the order of the goal moves—one needs to examine the start state and possible solution paths to determine the order of the goal moves.

PA and ITT were computed as an overall measure, as well as separately for each of the four categories of problems characterised by levels of GA and SD: GA_High_ SD_High_, GA_High_ SD_Low_, GA_Low_ SD_High_, and GA_Low_ SD_Low_ (Fig. 2).

#### Temporal-order judgement task

The efficiency of millisecond TIP was assessed with temporal resolution and sequencing abilities using the temporal-order judgement task for the psychophysical assessment of auditory temporal-order threshold (ATOT) which was used in our previous studies [e.g.,^26,32,37,38^]. In it, participants are presented with sequences of paired clicks (rectangular pulses, each of 1 ms duration), delivered in rapid succession monaurally (i.e., the first click is heard in the left ear followed by the second click in the right ear, or vice versa). The clicks were generated with a Realtek ALC3246 sound controller and Waves MaxxAudio Pro software.

The task is to verbally report the order of clicks presented within each pair (i.e., left–right or right–left). The two clicks within each pair are separated by different inter-stimulus intervals. The task consists of two parts. In Part 1, 20 trials are performed. The stimuli within each pair are separated by fixed inter-stimulus intervals, changing in steps of 17 or 18 ms, ranging from 160 to 1 ms, in a decreasing (*n* = 10) and then increasing (*n* = 10) order. In Part 2, the initial inter-stimulus interval value is computed on the basis of the correctness achieved in the 20 trials performed in Part 1. Next, inter-stimulus interval values are generated by an adaptive maximum-likelihood algorithm^25,26,39–42^. Part 2 consists of 50 trials. The inter-stimulus interval in each trial is adjusted depending on the correctness achieved in the previous response. On the basis of the 70 trials completed in Parts 1 and 2, the ATOT is calculated (in milliseconds) for each participant^37^. The ATOT reflects the index of millisecond TIP efficiency and is defined as the shortest time gap between two clicks presented in rapid succession for which a participant could identify their temporal order (i.e., the before–after relation) with at least 75% correctness^40–45^.

To focus the participants’ attention, a warning signal was delivered binaurally 1 s before the first click within each pair. Prior to the task proper, participants performed a few practice trials with relatively long inter-stimulus intervals, and, after each response, feedback on the correctness achieved was given, in order to familiarise participants with the measurement procedure. Then the experiment proper started and no feedback on correctness was given.

### Statistical analyses

Prior to any analyses, the data were screened for outliers by converting them to *z*-scores using an exclusion criterion of *z* > 3 and *z* < − 3. Three participants were excluded from the original sample of 204, based on this procedure. All analysed data indicated no severe violation of normality^46^.

#### Age-related differences

To confirm age-related differences in TOL**–**F and in temporal-order judgement task, independent samples *t*-tests were performed, followed by Pearson correlations to examine the relationships between outcome measures from these two tasks in each group separately.

#### Testing for the proposed model

To evaluate if age moderates the relationship between planning and TIP, four separate conditional process analyses were conducted on categories considering structural problem properties in TOL**–**F, namely: (1) GA_High_ SD_High_, (2) GA_High_ SD_Low_, (3) GA_Low_ SD_High_, and (4) GA_Low_ SD_Low_. This framework analysis allows us to simultaneously test for moderation and mediation effects. ATOT was the predictor (i.e., the independent variable), PA the dependent variable, ITT the mediator of the PA–ATOT relationship, and age group was the moderator of the PA-ATOT and ITT-ATOT relationships (for the conceptual model see Fig. 1).

In this model there were two effects of interest: (1) the conditional direct effect of ATOT on PA, indicated by a significant ATOT x age group interaction, and (2) the conditional indirect effect of ATOT on PA via ITT, indicated by a significant index of moderated mediation (IMM). The significance of the conditional indirect effect is confirmed if the 95% bias-corrected bootstrap confidence interval (10,000 bootstrap resamples) for the IMM does not contain zero^33,47^. The analyses were run using Model 8 from the PROCESS macro^48^ in IBM SPSS 26.

## Results

### Age-related differences

#### TOL–F

For PA, significant differences between groups were observed for overall performance as well as for particular categories. Young participants achieved more points, reflecting more problems solved (better performance) than elderly participants. These relations are presented in Table 1.

**Table 1:** Mean (and SD) of planning accuracy (PA, in points) and initial thinking time (ITT, in seconds) for overall performance and for particular categories of TOL**–**F in the two age groups. Significant differences between groups (bolded) are marked by asterisks: **p < 0.01; ***p < 0.001.

	PA mean (SD)			ITT mean (SD)		
	Age group			Age group		
	Young (*n* = 110)	Elderly (*n* = 91)	Differences between groups (*t*-values)	Young (*n* = 110)	Elderly (*n* = 91)	Differences between groups (*t*-values)
**OVERALL**	16.7 (3.2)	12.8 (2.7)	9.2***	10.3 (4.2)	8.5 (3.5)	3.3***
CATEGORIES						
GA_High_ SD_High_	4.1 (1.2)	3.1 (1.2)	5.8***	10.7 (5)	9 (4.4)	2.6**
GA_High_ SD_Low_	3.7 (1.2)	2.8 (1.2)	7.2 ***	11.3 (5.4)	8.5 (3.4)	4.5***
GA_Low_ SD_High_	4.6 (1.1)	3.4 (1.2)	7.21***	9.5 (4.2)	8.9 (3.8)	1.2
GA_Low_ SD_Low_	4.4 (1.1)	3.5 (1)	6.4 ***	9.8 (4.8)	7.6 (3.9)	3.6***

For ITT, the group differences were significant for overall performance as well as for three categories: GA_High_ SD_High_, GA_High_ SD_Low_, and GA_Low_ SD_Low_. This reflects longer ITT in young than in elderly participants. These differences were nonsignificant for the GA_Low_ SD_High_ category (Table 1).

#### Temporal-order judgement

For ATOT, significant differences between groups were observed, *t*(170,1) = − 10.4; *p* < 0.001; *d* = − 1.6; *CI* = [− 41, − 27.9]. ATOT values were lower in young (*M* = 41.4 ms, *SD* = 20.4) than in elderly participants (*M* = 75.9 ms, *SD* = 25.7), reflecting better performance in the former group.

### Correlation analysis

**Figure 3:**
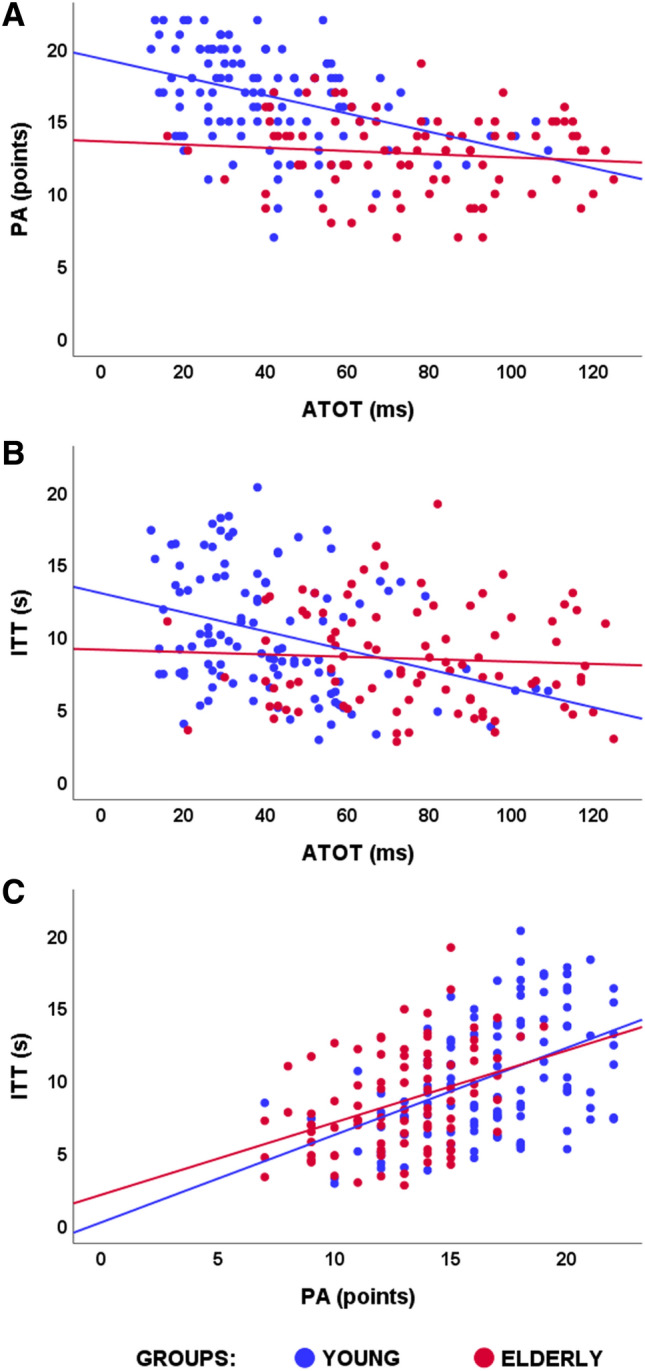
Scatter plots illustrating correlations between particular outcome measures of TOL–F and temporal-order judgement tasks in the two age groups: (**A**) between PA and ATOT, (**B**) between ITT and ATOT, (**C**) between PA and ITT. For more results, see Table 2.

In the young group, moderate significant correlations were found between PA and ATOT, and between ITT and ATOT, as well as between PA and ITT. Higher PA and longer ITT were accompanied by lower ATOT values (better performance). On the other hand, in the elderly, a significant correlation was observed only between PA–ITT. These results are presented in Table 2 and in Fig. 3.

**Table 2:** Pearson correlations coefficients between particular outcome measures of TOL–F and temporal-order judgement tasks. Significant correlations are marked with asterisks: **p < 0.01; ***p < 0.001.

	Young (*n* = 110)		Elderly (*n* = 91)	
	PA	ITT	PA	ITT
PA		0.47***		0.38***
ATOT	− 0.4***	− 0.32***	**− **0.11	**− **0.06

### Conditional process analyses for particular TOL–F categories

#### Category GA_High_ SD_High_

As shown in Table 3, age group moderated both the direct effect of ATOT on PA and the indirect effect of ATOT on PA via ITT. In the former case, it was reflected in the significant ATOT x age group interaction (*B* = 0.0224; *p* = 0.002) resulting from the significant effect of ATOT on PA in the young group (*B* = − 0.0207; *p* = 0.0002), being nonsignificant in the elderly group.

**Table 3:** Category GA_High_ SD_High_: summary of results of conditional process analysis. Abbreviations: *B* = unstandardised regression coefficient; *SE* = standard error; *t* = *t*-statistic value; *CI* = confidence interval; *LL* = lower limit, *UL* = upper limit. Significant results are marked with asterisks: *p < 0.05; **p < 0.01; ***p < 0.001. For the conditional indirect effects, the bootstrap-generated CIs are presented and significant effects are bolded.

Effect	*B*	*SE*	*t*	*p*	Bootstrapped 95% *CI*	
					*LL*	*UL*
**Mediator variable model (ITT as the outcome measure)**						
Constant	17.8011***	2.4781	7.1834	0.0000	12.9142	22.6881
ATOT	− 0.1437**	0.0467	− 3.0771	0.0024	− 0.2358	− 0.0516
Age group	− 3.7335*	1.7935	− 2.0817	0.0387	− 7.2705	− 0.1965
ATOT * age group	0.0630*	0.0284	2.2152	0.0279	0.0069	0.1190
**Dependent variable model (PA as the outcome measure)**						
Constant	6.3443***	0.6940	9.1419	0.0000	4.9757	7.7129
ATOT	− 0.0432***	0.0119	− 3.6210	0.0004	− 0.0667	− 0.0197
ITT	0.0471**	0.0178	2.6493	0.0087	0.0120	0.0821
Age group	− 1.8996***	0.4520	− 4.2026	0.0000	− 2.7911	− 1.0082
ATOT * age group	0.0224**	0.0072	3.1297	0.0020	0.0083	0.0366
**Conditional direct effect**						
Young	− 0.0207***	0.0055	− 3.7500	0.0002	− 0.0316	− 0.0098
Elderly	0.0017	0.0047	0.3701	0.7117	− 0.0075	0.0109

	*B*	*SE*	*LLCI*	*ULCI*		
**Conditional indirect effect**						
Young	− **0.0038**	**0.0017**	− **0.0076**	− **0.0010**		
Elderly	− 0.0008	0.0008	− 0.0027	0.0007		
**Index of moderated mediation**						
Index	**0.0030**	**0.0017**	**0.0003**	**0.0070**		

Similarly, age group moderated the indirect effect of ATOT on PA via ITT (the confidence interval of the IMM did not contain zero: *IMM* = 0.0030, *CI* = [0.0003, 0.0070]). In the young group, a significant effect was found (*B* = − 0.0038, *CI* = [− 0.0076, − 0.0010]), being nonsignificant in the elderly.

#### Category GA_High_ SD_Low_

Age group did not moderate the direct effect of ATOT on PA (Table 4). In contrast, the indirect effect of ATOT on PA via ITT was significant and moderated by age group (*IMM* = 0.0048, *CI* = [0.0014, 0.0098]). A significant effect was found in the young group (*B* = − 0.0051, *CI* = [− 0.0098, − 0.0018]), whereas, it was nonsignificant in the elderly.

**Table 4:** Category GA_High_ SD_Low_: summary of results of conditional process analysis. Abbreviations: *B* = unstandardised regression coefficient; *SE* = standard error; *t* = *t*-statistic value; *CI* = confidence interval; *LL* = lower limit, *UL* = upper limit. Significant results are marked with asterisks: *p < 0.05; **p < 0.01; ***p < 0.001. For the conditional indirect effects, the bootstrap-generated CIs are presented and significant effects are bolded.

Effect	*B*	*SE*	*t*	*p*	Bootstrapped 95% *CI*	
					*LL*	*UL*
**Mediator variable model (ITT as the outcome measure)**						
Constant	21.0612***	2.3936	8.7990	0.0000	16.3409	25.7815
ATOT	− 0.1716***	0.0451	− 3.8030	0.0002	− 0.2605	− 0.0826
Age group	− 6.1406***	1.7324	− 3.5446	0.0005	− 9.5570	− 2.7242
ATOT * age group	0.0836**	0.0274	3.0467	0.0026	0.0295	0.1378
**Dependent variable model (PA as the outcome measure)**						
Constant	4.1572***	0.7322	5.6779	0.0000	2.7133	5.6012
ATOT	− 0.0157	0.0121	− 1.2969	0.1962	− 0.0396	0.0082
ITT	0.0576**	0.0185	3.1187	0.0021	0.0212	0.0940
Age group	− 0.7333	0.4631	− 1.5835	0.1149	− 1.6466	0.1800
ATOT * age group	0.0056	0.0073	0.7694	0.4426	− 0.0088	0.0200
**Conditional direct effect**						
Young	− 0.0101	0.0056	− 1.8060	0.0725	− 0.0211	0.0009
Elderly	− 0.0045	0.0047	− 0.9632	0.3366	− 0.0137	0.0047

	*B*	*SE*	*LLCI*	*ULCI*		
**Conditional indirect effect**						
Young	− **0.0051**	**0.0020**	− **0.0098**	− **0.0018**		
Elderly	− 0.0002	0.0008	− 0.0020	0.0014		
**Index of moderated mediation**						
Index	**0.0048**	**0.0021**	**0.0014**	**0.0098**		

#### Categories GA_Low_ SD_High_ and GA_Low_ SD_Low_

In these two categories, both the direct and indirect conditional effects of ATOT on PA were nonsignificant.

## Summary of results

We confirmed the age-related declines in both planning (for overall performance and particular categories of problems; Table 1) and TIP. Despite significant correlations between the two TOL–F indicators (PA and ITT) in both age groups, these indicators correlated significantly with ATOT only in the young group (Table 2, Fig. 3).

Conditional process analyses showed a differential effect of ATOT on PA, depending on the age group and category of problems (Tables 3, 4). The contribution of ATOT to PA was significant in the young group, but only in problems with GA_High_ (categories GA_High_ SD_High_ and GA_High_ SD_Low_) and not in problems with GA_Low_. For this contribution, the direct effect of ATOT on PA was found only in the category characterised by high levels of both GA and SD. The indirect effect of ATOT on PA (via ITT) was found in both categories of GA_High_ (i.e., GA_High_ SD_High_ and GA_High_ SD_Low_), thus it was independent of SD level.

## Discussion

The results of our study confirmed age-related declines in both planning and TIP, which have also been demonstrated in previous studies^25,49^. As mentioned in the Introduction, executive deficits are commonly linked to structural and functional changes in the prefrontal cortex^7^, whereas, the age-related deficits in the temporal resolution reported here may be explained by the processing-speed theory which explains how many distinct influences contribute to the relations between age and cognitive functioning^50^. It suggests that in advancing age, the reduction in processing speed leads to impairment of cognitive functions because of two presumable mechanisms, i.e., the limited time or simultaneity mechanisms. Accordingly, in elderly information processing is slowed down, thus, cannot be successfully completed within a limited time for such processing (termed as limited time mechanism), or delays at early stages of processing lead to reduced amount of information available for the later stages causing difficulties in efficient processing (the simultaneity mechanism)^50^. Based on the Salthouse’s theory it may be assumed that the temporal resolution, as measured by the ATOT (i.e., the minimum time gap separating two stimuli in rapid sequences) could reflect the age-related processing speed deficit.

In case of the lower ATOT the incoming information is chunked (and processed) in shorter units, whereas, the higher ATOT values correspond to longer processing units. As a consequence, in the former situation information can be efficiently processed in shorter time windows (more efficient limited time mechanism), as opposed to the latter situation when these processing windows are longer (less efficient mechanism). On the other hand, the lower ATOT values foster more chunks of information (rich information) available for processing at the later stages (more efficient simultaneity mechanism). In case of the higher ATOT because of longer chunks, the information cannot be completed at lower stages causing deficient processing at the later stages because of reduced available traces.

The support for the above assumption comes from studies pointing to the link between temporal resolution and processing speed. These studies showed that both of them are related to more efficient cognitive functions, including working memory or language abilities, especially to processes which require the coordination of mental operations^23,27^.

The deficient temporal resolution, therefore, affects particular cognitive functions that require highly dynamic processing associated with manipulation of processed material. In contrast, this has little to do with cognitive stability, reflected in maintaining elements in working memory. As the PA requires dynamic manipulation of processed material, its reduced efficiency in elderly was reflected in overall performance on TOL**–**F (in both PA and ITT; Table 1). Such age-related disruption of executive processing and TIP was also reflected in significant correlations between particular outcome measures of TOL**–**F and TIP (Table 2, Fig. 3a,b) that were significant only in the young group (*n* = 110). These correlations in the elderly (*n* = 91) were nonsignificant. Such divergent age-related relationships are further explained by the conditional process analyses discussed below. On the other hand, the correlations between the two TOL**–**F outcome measures (PA and ITT) were significant in both groups, indicating that better PA corresponds to longer duration of the preplanning phase (ITT; Fig. 3c). This suggests the internal consistency of the test performance, independently of the age group.

Due to the multiparameter structure of TOL**–**F, we found that the structural properties of problems affect the relationship between planning and TIP in the two age groups studied here. Accordingly, in the young group, TIP predicted planning only in categories characterised by GA_High_, regardless of SD level (SD_High_ or SD_Low_; Tables 3, 4). This effect may be explained by the nature of the mental processes engaged in GA_High_ problems, which involve situations where the order of the subgoals cannot be straightforwardly inferred from the goal state^11^. This necessitates flexible shifts of mental operations enabled by disengaging from previously selected paths. Therefore, performance of GA_High_ tasks places high demands on cognitive flexibility, which entails flexible changes in working memory representations^11^. As these operations are characterised by a specific temporal dynamics, skilled millisecond TIP associated with the high temporal resolution may foster optimal processing.

The question is why TIP appears to contribute to planning only in the young group. The answer may lie in the nature of the cognitive processes involved in TOL–F, namely working memory capacity (reflected in SD) and cognitive flexibility (reflected in GA). Both of these functions show a decline in advancing age^51,52^. However, the existing literature on task switching and set shifting (often interpreted as measures of cognitive flexibility) suggests that the age-related deficit is caused not by the slowing of the switch itself, but by more fundamental difficulties in maintaining several alternative sets of representations in working memory^53–55^ preventing the degraded information from being further processed successfully. Thus, it can be hypothesised that difficulties in solving cognitively demanding problems (characterised by either GA_High_ or SD_High_) are rooted in working memory deficits—in other words, they reflect working memory capacity rather than processing efficiency. These maintenance problems seem to be associated with age-related deterioration in cognitive functions, independently of TIP resources. Therefore, efficient TIP may not support TOL-F performance in elderly. On the other hand, in young participants such maintenance resources work at an optimal level. We can assume that their performance in GA_High_ problems reflects their cognitive flexibility, which is related to the efficiency of TIP.

In the conditional process analyses, we indicated that TIP influences planning in two ways: directly (adjusting cognitive processing of planned execution) and indirectly (through enabling more efficient use of the preplanning strategy reflected in ITT, see Fig. 1). Indeed, the obtained results show that, depending on the age group, preplanning mediates the relationship between TIP and planning (Tables 3, 4). In young adults, better temporal resolution fosters more effective planning during the relatively long preplanning phase (Table 1), in which mental operations are segmented into smaller chunks to be more effectively processed (Table 2, Fig. 3). Hence, in young adults, the longer ITT allowed the construction of a more effective plan during the preplanning phase. In contrast, in elderly people, the reduced ITT duration (Table 1) results in less effective preplanning. This may suggest that, because of this incomplete preplanning, the elderly participants support their performance with online planning during the execution phase, which appears to be a less effective strategy. This indirect effect of ATOT on PA via ITT was present in categories characterised by GA_High_, independently of the SD level.

It should be stressed that the direct effect of ATOT on PA was observed only in one category: GA_High_ SD_High_ (Table 3). In these tasks, more demands seem to be placed on working memory due to the high SD level. This requires holding a goal move in mind while planning a sequence of moves, taking into account the interdependencies of the moves (i.e., situations where one move influences the possibility of another move^11^). In SD_High_ problems, the cognitive load on working memory is high not only during the mental preparation phase, but also during the execution phase. These problems, therefore, involve not only efficient preplanning, but also the dynamic updating of the performed sequence of moves (due to the interdependencies) and the implementation of a plan without mistakes, which can be supported by efficient TIP.

This study draws novel conclusions on the topic of planning, its relationship with TIP moderated by age, and the effect of two structural properties (SD and GA) on the cognitive processes underlying planning. In a sample of 201 young and elderly adults, we showed that TIP influences planning only on tasks characterised by GA_High_, which we explained by the nature of the dynamic mental operations engaged. We proposed a possible explanation as to why this relationship was observed only in the young group. As planning relies on both working memory capacity (static maintenance reflected in SD) and dynamic cognitive flexibility (reflected in GA), the divergent effect of aging reported here should be further studied, as this would expand our knowledge of the complex phenomenon of age-related deterioration in planning.

## Data Availability

The datasets generated and/or analysed during the current study are available from the corresponding author on request.

## Abbreviations

ATOT: Auditory temporal-order threshold
GA: Goal ambiguity
IMM: Index of moderated mediation
ITT: Initial thinking time
PA: Planning accuracy
SD: Search depth
TIP: Temporal information processing
TOL**–**F: Tower of London**–**Freiburg version
